# Selected Elements of the Tumor Microenvironment (MMP-2, MMP-7, TIMP-2, CXCL-9, CXCL-10) in the Serum of Pediatric Patients with Acute Lymphoblastic Leukemia

**DOI:** 10.3390/cells14040297

**Published:** 2025-02-17

**Authors:** Aleksandra Kaczorowska, Natalia Miękus-Purwin, Anna Owczarzak, Anna Gabrych, Małgorzata Wojciechowska, Ninela Irga-Jaworska, Sylwia Małgorzewicz, Małgorzata Rąpała, Joanna Stefanowicz

**Affiliations:** 1 Department of Pediatrics, Hematology and Oncology, University Clinical Center of Gdansk, Debinki 7, 80-211 Gdańsk, Poland; miekus@gumed.edu.pl (A.K.); ankagabrych@gumed.edu.pl (A.G.); wojciechowska88@gumed.edu.pl (M.W.); nirga@gumed.edu.pl (N.I.-J.); 2 Independent Researcher, 81-553 Gdynia, Poland; miekusn@gmail.com; 3 Division of Clinical Nutrition and Dietetics, Faculty of Health Sciences with the Institute of Maritime and Tropical Medicine, 7 Dębinki, 80-211 Gdańsk, Poland; anna.owczarzak@gumed.edu.pl (A.O.); sylwia.malgorzewicz@gumed.edu.pl (S.M.); 4 Department of Paediatrics, Haematology and Oncology, Faculty of Medicine, Medical University of Gdansk, 7 Debinki Street, 80-210 Gdansk, Poland; 5 Department of Pediatric Surgery, Marciniak Hospital, 2 Gen. Augusta Emila Fieldorfa, 54-049 Wroclaw, Poland; mrapala@poczta.onet.pl; 6 Faculty of Health Sciences, Medical University of Gdansk, 3a Maria Sklodowska-Curie Street, 80-210 Gdansk, Poland

**Keywords:** tumor microenvironment, metalloproteinases, tissue inhibitors of metalloproteinases, cytokines, chemokines, pediatric cancer, leukemia

## Abstract

In recent years, researchers have been paying special attention to the tumor microenvironment (TME). One of the most important factors contributing to the development and progression of cancer is the destruction of elements of the extracellular matrix (ECM). The most important substances involved in regulating the extracellular matrix degradation process are extracellular matrix metalloproteinases (MMPs) and their inhibitors (TIMPs). In the process of cancer cell migration, chemokines secreted by target tissues, as well as the profile of chemokine receptors presented on cancer cells, play a key role. In the presented work, five components of the TME were selected: MMP-2, MMP-7, TIMP-2, CXCL-9, and CXCL-10. In the years 2018–2021, peripheral blood samples were collected before the start of treatment and then on day 33 of intensive treatment from 31 patients diagnosed with ALL. The results indicate that the levels of MMP-2, MMP-7, and TIMP-2 did not statistically significantly change before and during treatment of ALL patients. The decrease in CXCL-9 and CXCL-10 levels in the patients’ serum on the 33rd day of therapy turned out to be statistically significant. Our study indicates that serum is also a valuable material for the evaluation of these substances. Conclusions: CXCL-9 and CXCL-10 could be used as one of markers for monitoring the response to treatment and a potential marker of ALL recurrence in pediatric patients. The role of MMP-2, MMP-7, and TIMP-2 in the assessment of response to therapy in children with ALL has not been confirmed.

## 1. Introduction

### 1.1. Leukemia and Its Treatment

According to the newest National Cancer Institute statistics, childhood leukemia represents 25.4% of all new childhood cancer cases, making it the most common type of pediatric malignancy. It is most frequently diagnosed among children aged 1–4 years [1]. In the group of childhood leukemias, acute lymphoblastic leukemia (ALL) and acute myeloid leukemia (AML) are the most common types, with ALL accounting for over 80% of all cases. ALL develops as a consequence of malignant transformation of a single abnormal progenitor cell that has the capability to expand (in a so-called clone of similar progeny cells) by indefinite self-renewal. The events that lead to malignant transformation are complex and multifactorial [2]. The unregulated growth of clonal lymphoid cells most commonly concerns pre-B cells (80–85%), and less commonly T (10–15%) and mature B cells (<5%) [3]. Stabilized leukemia incidence is observed in children aged 0–14 years, while in the group aged 15–19 years, leukemia incidence rates are still slowly increasing. In Cancer Statistics 2024, based on diagnoses from previous years and the follow-up of all patients through 2020, it is predicted that overall survival (OS) in leukemias will reach 88% (patients 0–14 years old) and 77% (patients 15–19 years old) [4]. Boys with childhood ALL have experienced inferior outcomes compared to girls, and it is believed to be attributable to increased CNS relapses in boys [5].

Despite an increasingly individualized therapeutic approach, there is still a group of patients in whom disease relapses and resistance to treatment is observed. This calls for further attempts to understand the mechanisms of leukemogenesis, as it may be possible to discover new potential targets for therapy.

It is certain that leukemogenesis is a multi-stage process, and the cause of leukemia is multifactorial. Over the last five decades, we witnessed great advances in understanding the genetic and biological basis of childhood ALL. Leukemic cells are being investigated by methods ranging from karyotyping (which identifies large chromosomal alterations) to whole genome sequencing (which identifies cryptic changes in the entire genome) [6]. Genomic analyses have led to creating more accurate risk-stratification groups and—in some cases—targeted therapy but did not establish the basis of ALL in a considerable group of patients [7]. Several lines of investigation indicate that a subset of childhood leukemia cases arise before birth. Specific chromosomal translocations predisposing to pediatric B-cell ALL may be detected at birth in blood spots and cord blood years before the clinical onset of leukemia, generating clinically silent pre-leukemic phase [7,8]. Furthermore, new data suggests that children with ALL are born with a dysregulated immune function, which—together with post-natal environmental exposure—causes childhood ALL. Soegaard et al. measured concentrations of selected cytokines and other inflammatory markers in neonatal blood spots obtained from 178 children who were later diagnosed with B-cell precursor ALL. Their results support this hypothesis [9]. It is crucial to keep searching and identifying which factors are responsible for the long persistence of the pre-leukemic cells in individuals and for their eventual transformation into cancer cells [8].

Therapy for ALL is rapidly evolving, with the development of immunotherapies that target leukemia-specific antigens using unconjugated antibodies, antibodies conjugated to drugs or toxins, antibodies that link leukemia cells to effector cells, or engineered CARs. Key discoveries in the treatment of ALL in recent years include the use of Blinotumobab and CARTs. Blinatumomab is a BiTE (bi-specific T cell engager) that binds both CD3 and CD19, bringing T cells in proximity to CD19-expressing malignant and normal B cells, resulting in the perforin-mediated cytotoxic killing of CD19+ cells [10]. Blinatumomab is highly effective at clearing marrow MRD [11,12]. CAR-T-targeting CD19 are highly effective, with 80% to 90% of patients who have heavily pretreated, highly chemorefractory leukemia achieving MRD-negative remissions [13,14]. In a subset of patients, remissions are sustained without subsequent therapy, although, for many patients, CD19 CAR-T is employed as a bridge to HSCT as definitive therapy. Tisagenlecleucel (Kymriah) was approved by the FDA in 2018 for the treatment of B-ALL for children up to age 25 years who are in second or greater relapse, or who have refractory disease, based on an international phase 2 trial of 75 patients demonstrating remission rates of 81% at 3 months, with all responders having MRD less than 0.01%; 12-month EFS was 50% [15].

### 1.2. Tumor Microenvironment (TME), Bone Marrow Microenvironment (BMM)

Technological progress allows us to examine cancer cells and the environment surrounding them in increasingly greater detail. This “single cell” approach is crucial not only because blood cancers are a highly heterogenous group of diseases, but also because TME comprises heterogenous immune dynamics that may be different between patients [16]. It seems impossible to discuss the causes of ALL development and search for new treatment targets without considering the environment in which abnormal cells exist and the complicated interactions that occur in this environment. Emerging research has acknowledged the vital role of TME in treatment resistance and disease relapse [17]. The TME surrounding the cancer cell includes the extracellular matrix (ECM), blood vessels, fibroblasts, immune system cells, and numerous substances secreted by these cells. It has been proven that interactions between cancer cells and TME may contribute to the development, progression, and formation of cancer metastases, as well as the effectiveness of treatment [17,18]. It is observed that the efficacy of immunotherapy in leukemia remains limited due to factors such as the immunosuppressive TME and lack of suitable immunotherapeutic targets. Thus, an in-depth characterization of the TME in pediatric leukemia is warranted to improve the efficacy of immunotherapy [19]. As leukemias are considered “liquid tumors”, in which the bone marrow is the main maintenance site of malignancy, the term bone marrow microenvironment (BMM) is preferred. Liquid tumors are cancers that develop in the blood, bone marrow, or lymph nodes. Cancerous cells are present in body fluids and circulate through the body via the bloodstream or lymphatic system, rather than forming a solid mass.

The bone marrow is an organ with a complex structure, which contains both hematopoietic and non-hematopoietic cells. These non-hematopoietic cells not only physically surround hematopoietic cells, but also actively regulate hematopoietic processes through the secretion of cytokines, hormones, and growth factors [8]. Bone marrow provides a framework of microenvironmental niches that support the function of immune cells and hematopoietic stem cells (HSCs). The concept of bone marrow cellular niches was first formulated 30 years ago by Schofield [20]. The key role of these niches is to provide the most favorable conditions for HSCs, where they are protected from environmental stresses and receive adequate support for maintaining self-renewal and multi-lineage differentiation capacity [21]. Such conditions are provided in several different ways, for example, by soluble factors and cell–cell contact [22]. Because the hematopoietic and immune systems need to rapidly respond and adapt to the current needs of the organism, their niche within the bone marrow should not be viewed as a static entity, but rather as a microenvironment that continually processes and transports information [23]. To date, at least three distinct HSC niches have been widely described: endosteal, arteriolar, and sinusoidal. Each niche has defined characteristics and cellular types, detailed discussion of which is far beyond the scope of this article [24]. In healthy bone marrow, microenvironmental niches create the most favorable conditions for HSCs. In leukemia patients, leukemic stem cells (LSCs) actively interact with the BMM, causing its dysregulation [25]. In effect malignant niches are created; they become friendly to LSCs and unfriendly to healthy HSCs. This allows LSCs to survive and gain chemoresistance and leads to the persistence of the disease [17,21].

### 1.3. Chemokine Ligand 9 and 10 (CXCL-9, CXCL-10)

TME is the primary location in which tumor cells and the host immune system interact. Among many others, a key role in this process is played by chemokines and their receptors. Chemokines are soluble small proteins (8–12 KDa) that are divided into four sub-families, CC, CXC, CX3C, and XC, based on the differences in the configuration of the two (or one, in the case of XC) highly conserved cysteines located closest to the N-terminus. The cysteine residues are responsible for the disulfide bridges formation which are relevant to main structural integrity, which is a basis for further chemokine binding to its corresponding G-protein-coupled receptors (GPCRs) [26,27,28,29]. There are seven G-protein-coupled chemokine receptors. Specifically, chemokine C–X–C ligand 10 (CXCL10) and ligand 9 (CXCL9) are the members of the family of CXC chemokines and after binding to their corresponding receptor, CXCR3 demonstrate their biological functions [30,31]. Three CXCR3 variants are known: CXCR3-A, CXCR3-B, and CXCR3-Alt. CXCR3-A is mainly expressed on activated T and natural killer (NK) cells, whereas CXCR3-B is distributed on vascular endothelial cells [32]. CXCL11, CXCL10, and CXCL9 could bind either to CXCR3-A or CXCR3-B, and that way, drive the chemotaxis and proliferation of cells (while binding to CXCR3-A) or inhibit cell migration and proliferation and induce apoptosis (after binding to CXCR-3B). CXCR3-Alt binds only to CXCL11, and its specific role is still controversial [32,33]. The migration of leukocytes to the TME to exert not only their anti- but also pro-tumoral activity is mediated by the expression of CXCR3-A on their surface. The binding to CXCR3-A activates inhibitory G protein (Gi) and G protein q (Gq) and triggers their downstream signaling pathways, including the intracellular Ca^2+^ release, extracellular signal-regulated kinase (ERK1/2), and Akt (a serine threonine protein kinase downstream of the phosphoinositide 3-kinase (PI3K)) pathways. The scientific data revealed that the expression of CXCR3-A on malignant tumor cells (such as, i.e., glioblastoma, colorectal cancer, high-grade serous ovarian cancer, lung adenocarcinoma, and breast cancer) leads to an increased proliferation and spreading [34]. Since chemokines monomers and homodimers can interact (heterodimers and/or heterooligomers are formed) the relation between chemokine structure and function in not straightforward. Nevertheless, their involvement in innate and adaptive immune responses, immune surveillance, and pathological inflammation due to the influence on leukocytes development, differentiation, migration, and distribution was clearly verified in physiological and pathophysiological conditions. Chemokines have also been proven to regulate many other cellular processes due to their involvement in migration, proliferation, and survival in multiple cell types [26,27,28,29]. Cytokine and chemokine signaling are essential for BMM. Studies on relapsed ALL patients led to the discovery that the expression of a broad spectrum of cytokines and their receptors were higher than of healthy BM samples [21].

### 1.4. Matrix Metalloproteinases (MMPs) and Tissue Inhibitors of Metalloproteinases (TIMPs)

Every organ has a unique composition of the extracellular matrix (ECM) to serve a particular tissue specific purpose. The balance between the degradation and synthesis of ECM components is crucial to maintain homeostasis and the adequate functioning of the tissue. The TME-associated ECM is essentially different from that of healthy tissue stroma, as the changed expression of matrix-remodeling enzymes leads to abnormal ECM dynamics. One of the key steps leading to the growth and spread of cancer is the degradation of the ECM, and the most important substances involved in this process are matrix metalloproteinases (MMPs) and their inhibitors (tissue inhibitors of metalloproteinases, TIMPs).

According to the available data, MMP-2 and MMP-7 are among the most important metalloproteinases in the pathogenesis of tumor formation. The involvement of these metalloproteinases in cancer development has been repeatedly confirmed. The most important studies available in the literature on this subject were discussed in our previous review article [18]. However, the studies have been mainly conducted on the adult population, with the majority of reports being conducted on solid tumors. In the pediatric population, most reports on the role of MMPs in cancer development also concern solid tumors, and relatively few studies have investigated the role of MMPs and TIMPs in non-solid tumors, such as leukemias [18,35].

### 1.5. Aims of the Study

The study aimed to answer the questions:

1. Whether the levels of MMP-2, MMP-7, TIMP-2 and chemokines CXCL-9 and CXCL-10 in the serum are impaired in pediatric patients with ALL,

and thus:

2. Whether they could serve as markers for assessing response to treatment;
3. Whether treatment can be monitored on their basis;
4. Whether the peripheral blood of ALL patients—as a biological material that is much more accessible—will reflect the results obtained from the bone marrow.

## 2. Materials and Methods

### 2.1. Sample Collection

In the years 2018–2021, peripheral blood was collected from 31 pediatric patients with newly diagnosed ALL at the Department of Pediatrics, Hematology, and Oncology of the Medical University of Gdansk and University Clinical Centre in Gdansk. The characteristics of the group are presented in Table 1 and Table 2. All examined patients were Caucasian, came from the same region of Poland, and did not show any significant socioeconomic differences. Abnormalities in physical examination at admission were mostly typical for the diagnosis of ALL and included: hepatosplenomegaly (27/31), paleness (23/31), lymphadenopathy (21/31), petechiae (9/31). In the three patients we observed tonsil enlargement. One of the patients presented swelling of the eyelid, and one presented swelling of the lower limbs.

Blood samples were collected before treatment (day 0) and then on the 33rd day of intensive treatment, according to the AIEOP-BFM POLAND 2017 protocol. In two patients, due to a pre-laboratory error, samples could not be secured on the 33rd day of treatment (Table 3). The blood was centrifuged immediately after collection, and serum was frozen at −80 °C. Only 4 out of 31 patients were MRD (+) on the 33rd day of treatment.

Additionally, the levels of the following parameters from peripheral blood were recorded both at day 0 and day 33 of treatment: hemoglobin [g/dL]; platelets [G/L]; white blood count (WBC) [G/L]; neutrocytes [G/L]; C-reactive protein (CRP) [mg/L]; lactate dehydrogenase (LDH) [U/L]; ferritin [ng/mL]; creatinine [mg/dL]; uric acid [mg/dL]; alanine aminotransferase (ALT) [U/L]; fibrinogen [G/L]; prothrombin index (%); D-dimer [µg/L FEU]. These laboratory tests were performed in Central Clinical Laboratory of the Medical University in Gdansk.

### 2.2. Laboratory Analysis

The levels of MMP-2, MMP-7, TIMP-2, CXCL9/MIG and CXCL-10/IP in the patient’s blood serum were determined by the immunoenzymatic method, enzyme-linked immunosorbent assay (ELISA), using Human Quantikine R&D kits, Minneapolis, MN, USA, according to the manufacturer’s instructions. Duplicate measurements were performed in all cases and all laboratory parameters measured were assayed for interference. No significant cross-reactivity or interference was observed. The test results were read using the BIO TEK 800/TS device (bio-TEK Instruments). Measurements were performed in the research laboratory in the Department of Clinical Nutrition, Medical University in Gdansk.

**Human CXCL 10/IP**—detection ranged from 0.41 to 4.46 pg/mL. The mean MDD was 1.67 pg/mL.

Intra-assay precision CV(%) = 3.3; inter-assay precision CV (%) = 5.2.

**Human CXCL 9**—the minimum detectable dose (MDD) of human MIG ranged 1.37–11.31 pg/mL. The mean MDD was 3.84 pg/mL. Intra-assay precision CV (%) = 3.1; inter-assay precision CV(%) = 6.2.

**Total MMP-2**—the minimum detectable dose (MDD) of MMP-2 ranged from 0.014–0.082 ng/mL. The mean MDD was 0.033 ng/mL. Intra-assay precision CV (%) = 3.6; inter-assay precision CV (%) = 7.0.

**Human TIMP-2**—the minimum detectable dose (MDD) of human TIMP-2 ranged from 0.004 to 0.064 ng/mL. The mean MDD was 0.011 ng/mL.

**Human Total MMP-7**—the minimum detectable dose (MDD) of human MMP-7 ranged from 0.005 to 0.094 ng/mL. The mean MDD was 0.016 ng/mL. Intra-assay precision CV (%) = 3.4; inter-assay precision CV (%) = 4.6.

### 2.3. Statistical Analysis

The results of the obtained studies were subjected to statistical analysis. The number of cases (N), median, range (min–max) and lower and upper quartiles (25Q–75Q) of the quantitative parameters studied were calculated for all groups. Quantitative data were presented as median, interquartile range M (25Q ÷ 75Q), and 95% confidence interval (95%CI). Normality of distribution was checked using the Shapiro–Wilk test, and homogeneity of variance was checked using Levene’s test. The percentage change in parameters was calculated according to the following formula: ΔX = 100 ∗ (X_2_ − X_1_)/X_1_ (where X_2_—parameter value on day 33 and X_1_—parameter value before treatment). The hypothesis of equality of median parameters in dependent groups was verified using the Wilcoxon nonparametric test. For selected pairs of parameters, correlation analysis was performed by calculating the Spearman correlation coefficient (R). *p* ≤ 0.05 was considered statistically significant. Statistical analysis was performed using the EPIINFO Ver. 7.2.3.1. and Statistica Ver. 13.3 computer statistical software package.

## 3. Results

**Figure 1:**
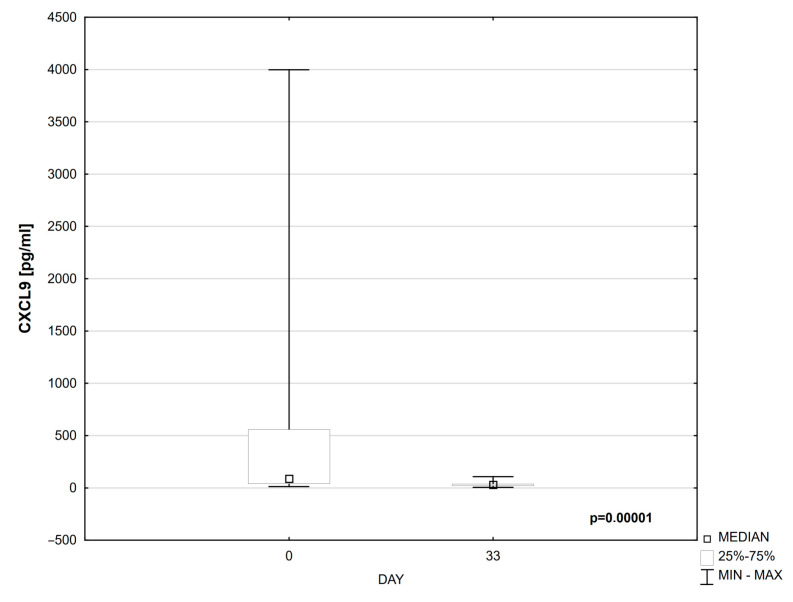
CXCL9 on days 0 and 33 of treatment.

**Figure 2:**
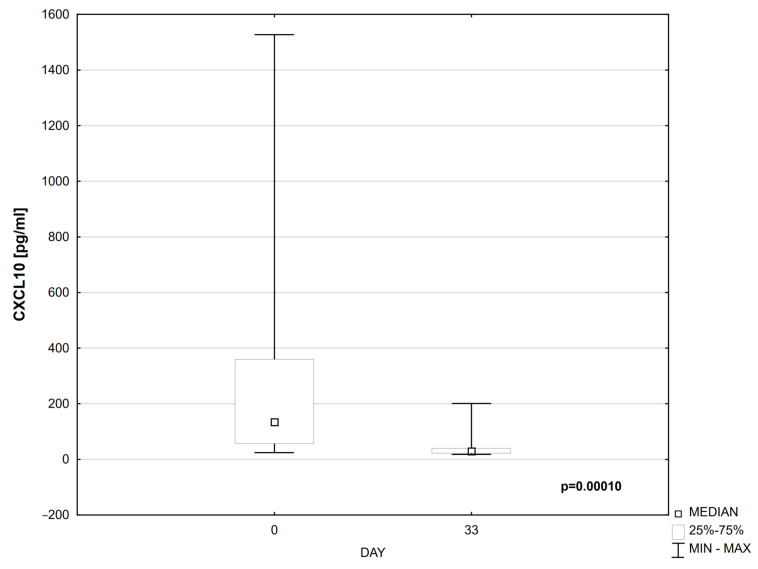
CXCL10 on days 0 and 33 of treatment.

Compared to day 0, a statistically significant decrease in the levels of CXCL-9 (*p* = 0.00001) and CXCL-10 (*p* = 0.00010) was observed in the serum of patients on day 33 of treatment (Table 4, Figure 1 and Figure 2).

MMP-2, MMP-7, and TIMP-2 levels did not change statistically significantly before and during the treatment of ALL patients (Table 4).

Additionally, the change in CXCL10 and MMP-2 levels on day 0 and day 33 almost correlates with the initial blastosis in the bone marrow (Table 5). The MMP-2 level and the change in MMP levels negatively correlate with the CRP level (Table 6).

**Figure 3:**
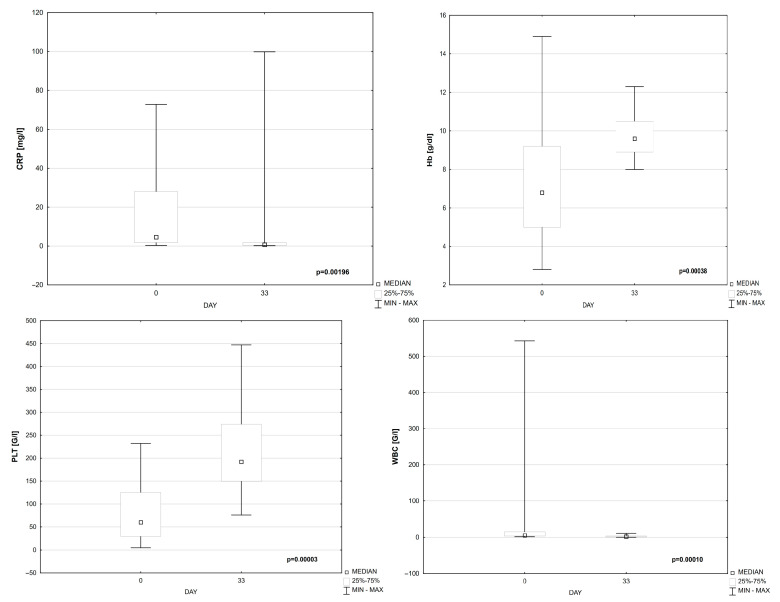
CRP, Hgb, PLT, and WBC on day 0 and 33 of treatment.

The significant changes in CRP, Hgb, PLT, and WBC on days 0 and 33 of treatment were observed (Table 4, Figure 3).

## 4. Discussion

### 4.1. CXCL-9 and CXCL-10

The role of inflammation in hematological malignancies is complex given that cells that mediate immune response are themselves cancerous. In cancer, cytokines families play a role in its growth, invasion, and metastasis. Current evidence suggests that both LSC and stromal cells secrete proinflammatory factors that actively suppress the function of healthy HSCs. In the bone marrow of AML patients, Chen et al. observed interferon gamma pathway activation, along with secretion of its chemokine target CXCL-10 [36]. Also in AML, in an experimental study, it was observed that the expression of the CXCL-10 gene (involved in angiogenesis) was increased in selected leukemia cell lines [37]. The potential of targeting these chemokines and their receptors to promote antitumor immune response in patients with cancer is discussed. Poor tumor infiltration by T cells has been attributed to potent epigenetic silencing of the genes encoding the Th1-type chemokines CXCL-9 and CXCL-10 in tumors [38,39]. Studies have shown that improved therapeutic responses to cancer immunotherapy and chemotherapy are associated with increased levels of TH1-type chemokines and increased numbers of effector T cells in TME. Therefore, cancer epigenetic reprogramming may remove the epigenetic repression of genes-encoding Th1-type chemokines, thus promoting effector T cell trafficking into the TME and improving the therapeutic efficacy of immunotherapy [40].

Scientific findings revealed that tumor cells express chemokines and/or chemokine receptors abnormally. In response to specific chemokines, different immune cell types migrate into the TME and regulate tumor immune responses. CXCL9 and CXCL10 are capable of chemoattracting the lymphocytes leading to the tumor-infiltrating lymphocytes (TILs) phenomena [31]. CXCL-9 and CXCL-10 are also known to be endogenous tumor angiogenesis inhibitors. Taking all this information into account, the conclusion is that chemokines directly and indirectly affect tumor immunity, shape its immune phenotype, and regulate tumor cell proliferation, invasiveness, and resistance to treatment [40].

Abnormalities of cytokines and growth factor signaling pathways are characteristic of all forms of leukemia [41]. Cytokine profiling performed in adult patients undergoing phase 1 clinical trial of NKTR-255 with CD19-22 CAR-T cell therapy for B-cell ALL showed significant increases in Il15 and chemokines CXCL-9 and CXCL-10 [42]. The results of Hohtari et al.’s studies on adult patients with ALL suggest that ALL BM has a unique immune cell composition that is also associated with clinical response to therapy [43]. CXCR3, the receptor for CXCL-10, is highly expressed in malignant B cells. Furthermore, some results strongly suggest a protumoral role for human monocytes in B cell precursor ALL, orchestrated by CXCL-10 and its effect on tumor cell migration and invasion [44].

Most of the studies that have confirmed the imbalance of chemokines and cytokines in leukemia patients so far have been based on biological material taken from a bone marrow biopsy. Magalhaes-Gama et al. evaluated the levels of selected immunological molecules in bone marrow plasma obtained from 47 pediatric patients with newly diagnosed B-cell ALL. They measured the levels at the time of diagnosis and at the end of induction therapy. The results demonstrated high levels of the chemokines CXCL-9 and CXCL-10 and cytokines Il-6 and Il-10 at the time of diagnosis, and a notable decrease at the end of the induction therapy [45]. Similar data were obtained from bone marrow by Kerr et al. Their results showed that low-risk patients presented higher levels of selected bone marrow soluble mediators when compared to high-risk patients. On day 15 of induction therapy, the data demonstrated an overall decrease in biomarkers (including CXCL-9 and CXCL-10), regardless of the risk group stratification. Additionally, the minimal residual disease (MRD)-positive subgroup of patients presented higher levels of CXCL-9 and Il-6 when compared to negative MRD (-) [46]. Gomez et al. compared the levels of chemokine receptors in bone marrow samples from 82 children with ALL at diagnosis versus 15 at relapses and quantified the levels of chemokines in central system fluid (CSF) samples. In general, their results showed that CXCR3 signaling in ALL may be multifunctional, and not only chemotactic for the localization of leukemic blasts in specific niches, but also providing chemoresistance, increasing the chances of relapses [47]. In 2022, Aref et al. named CXCL-10 as a novel biomarker for B-ALL’s response to induction chemotherapy, as their research demonstrated that bone marrow plasma levels of CCL-2, CXCL-9, and CXCL-10 at day 0 were significantly higher as compared to their levels at day 15. Moreover, the soluble chemokine biomarkers at day 0 were related to MRD detection and risk severity stages, and the best predictive one for MRD status was CXCL-10 [48].

Zhang et al. examined the peripheral blood of pediatric patients with ALL for intracellular cytokine profile of T cells. The results showed that in both CD4+ and CD8+ T cells of ALL patients, there is a dysregulation in the functionality of Th1 and Th2 cells with a gross reduction in Th1 cell populations and an expansion in Th2 [49]. ALL pediatric patients’ peripheral blood was also examined by Luczynski et al. and it confirmed T cell activation and Th2 predominance at the time of diagnosis and during remission induction [50]. Over time, subsequent peripheral blood tests in ALL patients showed the increased presence of CD4(+) CD25(+) Treg cells and the altered levels of secreted cytokines [51,52].

As for CXCL-9 and CXCL-10, when our team started presented study in 2018, we did not find satisfying data in the medical literature about the level of these chemokines in the peripheral blood of pediatric ALL patients. However, in 2023, Carvalho et al. presented their research on the systemic immunological profile of 20 children with B-cell ALL. In summary, on day 0 of treatment, they observed increased levels of CCL-2, CXCL-9, CXCL-10, IL-6, and IL-10 (as well as increased frequency of CD4+ and CD8+ T cells, among other cells), while on day 35 they observed an opposite profile to that on D0 [53]. Our results showed similar data. That proved not only that peripheral blood might also serve as a valuable material for assessing the immune status of patients with ALL but also indicated their great potential as serum biomarkers. The mechanism of therapy-mediated changes in the levels of CXCL-9 and CXCL-10 could relate to the type of medications used in treatment protocol. Steroid therapy is a crucial element of ALL treatment in the initial weeks after diagnosis; therefore, CXCL-9 and CXCL-10, as proinflammatory cytokines, will decrease their values.

Pediatric patients diagnosed with ALL remain under the strict supervision of oncologists both during intensive treatment and after the completion of oncological treatment. They have regular peripheral blood tests, in accordance with the therapeutic protocol. Therefore, monitoring the level of CXCL-9 and CXCL-10 in their serum would be safe and would not require additional visits. The rising of these cytokines’ levels could potentially indicate the early relapse of the disease or its resistance to treatment.

### 4.2. MMP-2, MMP-7, TIMP-2

The BMM supports the progression of cancer cells through BMM-derived proteases such as MMPs. At present, it is known that there is no expression of MMP-2 in healthy bone marrow mononuclear cells. It is also acknowledged that leukemic cells express MMP-2; however, the precise role of MMP expression in ALL is still not clear [54].

The role of MMP-2 (together with MMP-9 and TIMP-1) in the clinical progression of pediatric ALL was examined by Saleh et al. Bone marrow samples were obtained from 76 patients, and those with MMP-2 overexpression showed a significant increase in the BM blast cell count at D0 and at D15 of treatment, as well as a significant increase in MRD at D15, compared to those with MMP-2 low-expression. This indicates its potential use as a prognostic marker for poor outcome of the patients [55]. Similar research, but using plasma as biological material, showed that at diagnosis MMP-2, MMP-9, and TIMP-2 were lower than in control group, whereas TIMP-1 levels were alike. During and after the treatment, an increased level of MMP-2 compared to the level at diagnosis was found. The levels of TIMP-2 increased in the following points of the study. These data suggested a probable—but complicated—role in leukemogenesis [56]. Other studies have focused on the connection between MMP-2 and MMP-7 genotypes and the risk of childhood leukemia. Researchers from Taiwan concluded that although MMP-2 promoter genotypes play a minor role in the assessment of the risk of developing leukemia, the MMP-7 A-181G genotype may serve as a predictive biomarker for childhood ALL [35,57]. Interesting data were delivered by Lynch et al. In their research, two leukemic cell lines—K562 (more aggressive leukemia blasts) and HL-60 (less aggressive blasts)—were analyzed for expression of several MMPs and their inhibitors. The expression of MMP-2 and TIMP-2 was similar in both cell lines, but only more aggressive cells expressed MMP-7. Additionally, by inhibiting MMP-7, a 40% reduction in invasiveness was revealed. This study emphasized that MMP-7 may play an important role in leukemia cell invasion [58].

Our results showed that MMP-2, MMP-7, and TIMP-2 levels did not change statistically significantly before and during the treatment of ALL patients, although MMP-2 levels at day 0 and day 33 correlate with the initial blastosis in the bone marrow.

Since in solid tumors, an important stage of tumor growth and its ability to metastasize is the destruction of the extracellular matrix, it seems that metalloproteinases will play a greater role in the TME of solid tumors, in contrast to leukemias, where the cancer is already disseminated at the beginning of the disease. It can therefore be assumed that in leukemias, cytokine-mediated processes will be more important, leading to the replacement of healthy hematopoietic cells with cancer cells and their survival in the bone marrow niche.

### 4.3. The Future of ALL Treatment

The concept of pre-leukemic and leukemic cells survival and evolution are strictly dependent both on genetic lesions and on the external signals coming from the microenvironment, paving the way to a new idea of dual-targeting therapeutic strategy, comprising microenvironmental and leukemia [8]. Within HSCs, the accumulation of recurrent mutations initiates malignancy. Simultaneously, specific alterations of the niches contribute to LSCs survival and expansion, providing protection from chemotherapy, leading to relapse [59]. Recognizing that TME contributes to treatment failure or success has led to a recent shift in cancer therapy. In leukemia, targeting components of BMM could present a novel means of enhancing therapeutic effectiveness, particularly in children with high-risk leukemia [60]. A variety of approaches are currently being researched to target the microenvironment, which include cytokines/chemokines and their receptors, adhesion molecules, signal transduction pathways, and hypoxia-related proteins [60,61]. In the study of Mumme et al., single-cell analysis revealed the altered tumor microenvironments of relapsed and remission-associated pediatric AML [62]. Furthermore, it could improve the effectiveness of tumor-directed immunotherapy approaches, including anti-leukemia CAR-T cells [8]. In fact, recent reports highlight the importance of the tumor microenvironment (among other factors) in resistance and relapse after CAR-T cell therapy [63]. The hope is that integrating knowledge of how bone marrow niches contribute to hematological disease predisposition, initiation, progression, and responses to therapy into future clinical practice will likely improve the treatment of these disorders [59].

## 5. Summary

It is certain that BMM, with its niches, plays a key role in the persistence and transformation of preleukemic clones into fully leukemic cells. In this context, inflammation has been highlighted as a crucial microenvironmental stimulus that is able to promote genetic instability, leading to disease manifestation [8].

In solid tumors, the immune contexture has been shown to impact outcomes. In ALL, the immunological status of BM niche has not been exhaustively studied, even though increasing evidence suggests that the immune system contributes both to the development and outcome of leukemia. Given the success of novel immunotherapies in oncology, it is urgent to discover the immunological basis of ALL [43].

In BM niches, chemokines are important signals for regulating hematopoiesis. It is observed that leukemic cells alter chemokine pathways in BM niches to eventually unseat healthy stem cells from BM protective niches. Consequently, it is believed that their inhibition could offer valuable therapeutic targets [8].

## 6. Limitations of the Study

We realize that the group of patients is not very large, but it should be remembered that cases of cancer in children are rarer than in adults, and it is more difficult to collect a large group of patients. It should also be remembered that the condition of the child before the start of treatment does not always allow for its inclusion in the conducted study. Moreover, the patient’s guardians do not always give consent for such participation. The study we conducted was a pilot study, and the main limitations of the study seem to be relatively small number of patients included and absence of control group.

## 7. Conclusions

In the present study, we proved that induction therapy has corrected abnormal levels of chemokines CXCL-9 and CXCL-10. Thus, we believe that CXCL-9 and CXCL-10 could be used as markers for monitoring the response to treatment and a potential marker of ALL recurrence in pediatric patients. The role of MMP-2, MMP-7, and TIMP-2 in the assessment of responses to therapy in children with ALL has not been confirmed.

## Figures and Tables

**Table 1:** Characteristics of the patients involved in the study at diagnosis (D0).

Patient	Type of All	% of Blasts in Bone Marrow at Diagnosis	% of Blasts in Peripheral Blood at Diagnosis	Genetic Risk Factors	Significant Co-Existing Diseases
1	ALL B-common	77.3%	1.5%	-	Hypoimmunoglobulinemia IgM and IgA; NF-1 susp.
2	ALL B-common	39.1%	45.4%	-	-
3	ALL B-common	89.3%	30.9%	-	-
4	ALL B-common	97.8%	49%	-	-
5	ALL B-common	90.2%	10%	-	-
6	ALL B-common	72.9%	0.00%	-	-
7	ALL B-common	70%	80.8%	-	-
8	ALL B-common	61.8%	0.00%	-	-
9	ALL B-common	97.6%	68%	-	-
10	ALL B-common	81.8%	-	-	-
11	ALL B-common	79.4%	8.8%	-	pathological fracture of Th11; arterial hypertension
12	ALL B-common	82.8%	-	-	alopecia areata
13	ALL B-common	86.4%	14%	-	-
14	ALL B-common	90.5%	0.00%	-	-
15	ALL B-common	27.4%	0.00%	rearrangement of E2A gene	-
16	ALL B-common	89.2%	71%	-	-
17	ALL B-common	87.6%	2%	-	arterial hypertension
18	ALL B-common	84.8%	40%	Ikaros gene	-
19	ALL B-common	75%	-	-	-
20	ALL B-common	95%	-	-	-
21	ALL B-common	84%	76.2%	BCR/ABL (Ph+)	psychomotor hyperactivity
22	ALL B-common	38.9%	0.00%	-	-
23	ALL B-common	67.6%	0.00%	-	status after treatment of hemophagocytic syndrome
24	ALL B-common	57.8%	43.4%	-	-
25	ALL pre-B	94.7%	23.7%	-	-
26	ALL pre-B	86.8%	78%	rearrangement of the MLL gene	pneumonia
27	ALL pro-B	72.8%	41.3%	-	-
28	ALL T-cell	77%	19%	deletion of TP53; unbalanced, complex karyotype	pilonidal cyst of the intergluteal cleft (status post 3 surgeries)
29	ALL T-cell	29.9%	16.8%	-	-
30	ALL pre-T	86.4%	94%	-	-
31	ALL pro-T	92%	71%	-	-

**Table 2:** Summary of the characteristics of the patients involved in the study.

	N	Median	Minimum	Maximum	Lower Quartile	Upper Quartile
Age at diagnosis [years]	31	5.00	1.25	14.50	2.50	8.50
Sex [F/M]	13/18 (41.9%/58.1%)					

**Table 3:** Characteristics of patients on the 33rd day of treatment.

Patient	Day of Treatment	Response to Treatment	Risk Group	Significant Co-Existing Diagnosis at the Time of Sample Collection
1	33	Remission	IR	Neuropathy after VCR; steroid diabetes; arterial hypertension
2	33	Remission	SR	Neuropathy after VCR; steroid diabetes; arterial hypertension; stomatitis
3	33	Remission	IR	-
4	33	Remission	IR	Fournier’s syndrome
5	33	Remission	IR	-
6	33	Remission	IR	Anal fissure with proctitis; stomatitis; hepatomegaly; fever
7	33	Remission	IR	-
8	33	Remission	IR	-
9	33	Remission	SR	Arterial hypertension; constipation; parenteral nutrition; drug-induced adrenal insufficiency; hypertriglyceridemia
10	33	Remission	IR	Two anal fissures
11	33	(+) MRD	Early HR	-
12	33	Remission	IR	Drug-induced adrenal insufficiency
13	33	Remission	IR	-
14	33	Remission	IR	Drug-induced adrenal insufficiency; hypertriglyceridemia; UTI
15	33	Remission	IR	-
16	33	Remission	IR	Status post VOD
17	33	Remission	IR	-
18	33	(+) MRD	Early HR	-
19	33	Remission	IR	-
20	33	Remission	Early non-HR	Condition after lobectomy due to invasive pulmonary fungal infection
21	33	Remission	IR	-
22	33	Remission	IR	-
23	-	-	-	-
24	-	-	-	-
25	33	Remission	SR	Drug-induced adrenal insufficiency
26	33	Remission	IR	Drug-induced adrenal insufficiency
27	33	Remission	IR	-
28	33	(+) MRD	Early HR	Stomatitis
29	33	Remission	IR	Drug-induced adrenal insufficiency; hypertriglyceridemia; UTI
30	33	Remission	IR	Cholestasis
31	33	(+) MRD	Early HR	CVC infection of *E. coli* ethology

IR—intermediate risk, SR—standard risk, HR—high risk, MRD—minimal residual disease, VCR—vincristine, UTI—urinary tract infection, CVC—central vena catheter, VOD—venooclusive disease.

**Table 4:** Comparison of the tested parameters before treatment and on the 33rd day of treatment.

	Day 0			Day 33		P (Wilcoxon Test)
	N	M (25Q ÷ 75Q)	95%CI	M (25Q ÷ 75Q)	95%CI	
MMP2 [ng/mL]	29	292.0 (180.0 ÷ 514.0)	100.0–1265.0	396.0 (252.0 ÷ 696.0)	90.0–970.0	0.261
MMP7 [ng/mL]	29	2.18 (1.48 ÷ 4.36)	1.18–11.66	1.92 (1.4 ÷ 2.58)	0.84–19.88	0.469
TIMP2 [ng/mL]	29	210.0 (147.7 ÷ 296.0)	122.0–401.0	193.0 (152 ÷ 286.0)	58.0–505.0	0.475
CXCL9 [pg/mL]	29	87.9 (39.8 ÷ 390.6)	12.9–3997.5	29.6 (21.1 ÷ 36.2)	4.9–53.1	0.00001
CXCL10 [pg/mL]	29	134.3 (71.0 ÷ 259.8)	31.9–601.3	29.0 (22.2 ÷ 39.3)	18.3–192.2	0.00010
CRP [mg/L]	29	4.62 (1.81 ÷ 27.9)	0.40–70.27	0.700 (0.4 ÷ 1.79)	0.390–34.86	0.00196
Hgb [g/dL]	29	6.80 (5.0 ÷ 9.2)	2.80–12.30	9.60 (8.9 ÷ 10.5)	8.10–12.00	0.00038
PLT [G/L]	29	60.0 (30.0 ÷ 125.0)	8.0–222.0	192.0 (154 ÷ 274.0)	83.0–437.0	0.00003
WBC [G/L]	29	5.31 (3.41 ÷ 14.72)	1.81–218.02	2.09 (1.07 ÷ 3.34)	0.45–10.98	0.00010
Fibrynogene [G/L]	29	2.67 (2.44 ÷ 3.96)	1.59–5.83	1.10 (0.88 ÷ 1.34)	0.56–4.04	0.00019
Protrombine indexy [%]	29	94.0 (90.0 ÷ 101.0)	75.0–104.0	89.0 (82–96.0)	69.0–106.0	0.0716
AlAT [U/L]	29	13.0 (9.0 ÷ 23.0)	5.0–92.0	42.0 (34 ÷ 90.6)	13.0–132.0	0.00048

M (25Q ÷ 75Q)—median, interquartile range. 95%CI—95% confidence interval.

**Table 5:** Changes in MMP2, MMP7, TIMP2, CXCL9, and CXCL10 levels at day 0 and day 33, and its correlations with the initial blastosis in the bone marrow.

	ΔMMP2			ΔMMP7			ΔTIMP2			ΔCXCL9			ΔCXCL10		
	N	R	P	N	R	P	N	R	P	N	R	P	N	R	P
Age [years]	29	−0.14	0.459	29	−0.28	0.142	29	−0.28	0.134	29	0.05	0.784	29	0.05	0.802
% blasts in bone marrow	29	0.35	0.0588	29	0.28	0.148	29	0.02	0.906	29	−0.05	0.784	29	0.35	0.0643
% blasts in peripheral blood	25	0.23	0.268	25	−0.11	0.589	25	0.28	0.172	25	−0.03	0.904	25	0.20	0.326
CRP [mg/L]	29	0.43	0.0184	29	−0.02	0.911	29	0.11	0.569	29	−0.08	0.690	29	−0.06	0.765
LDH [U/L]	29	0.13	0.513	29	−0.22	0.253	29	−0.07	0.736	29	0.01	0.972	29	0.03	0.861
Uric acid [mg/dL]	29	0.29	0.132	29	0.00	1.000	29	−0.22	0.256	29	0.32	0.0931	29	0.28	0.136

**Table 6:** Correlations of MMP 2, MMP 7, TIMP 2, CXCL9, CXCL10 with age, % of blasts in bone marrow, and peripheral blood, CRP, LDH, and uric acid.

	MMP2			MMP7			TIMP2			CXCL9			CXCL10		
	N	R	P	N	R	P	N	R	P	N	R	P	N	R	P
Age [years]	31	0.24	0.190	31	−0.03	0.853	31	0.20	0.285	31	−0.02	0.931	31	−0.07	0.698
% blasts in bone marrow	31	−0.21	0.266	31	−0.21	0.248	31	−0.19	0.299	31	0.10	0.602	31	−0.31	0.0852
% blasts in peripheral blood	27	−0.18	0.380	27	0.33	0.0887	27	0.05	0.811	27	−0.02	0.923	27	−0.30	0.131
CRP [mg/L]	31	−0.39	0.0288	31	0.27	0.147	31	−0.21	0.265	31	0.07	0.706	31	−0.03	0.878
LDH [U/L]	31	0.14	0.451	31	0.17	0.351	31	−0.01	0.959	31	0.19	0.317	31	0.15	0.413
Uric acid [mg/dL]	31	−0.17	0.371	31	0.23	0.222	31	−0.09	0.620	31	−0.26	0.161	31	−0.27	0.144

## Data Availability

The original contributions presented in this study are included in the article. Further inquiries can be directed to the corresponding author.

## Abbreviations

The following abbreviations are used in this manuscript:

TME	Tumor microenvironment
ECM	Extracellular matrix
MMP-2	Matrix metalloproteinase-2
MMP-7	Matrix metalloproteinase-7
TIMP-2	Tissue inhibitor of metalloproteinases-2
G-PCR	G-protein coupled receptors
CXCL-9	Chemokine ligand 9
CXCL-10	Chemokine ligand 10
CXCR-3	CXC chemokine receptor-3
OS	Overall survival
ALL	Acute lymphoblastic leukemia
AML	Acute myeloid leukemia
BMM	Bone marrow microenvironment
ELISA	Enzyme-linked immunosorbent assay
NK	Natural killer cells
WBC	White blood count
CRP	C-reactive protein
LDH	Lactate dehydrogenase
ALT	Alanine aminotransferase
HSC	Hematopoietic stem cells
LSC	Leukemic stem cells
MRD	Minimal residual disease
